# Mr. Knipe, on Epilepsy

**Published:** 1801-06

**Authors:** William Knipe

**Affiliations:** Garstang


### *To the Editors of the Medical and Phyſical Journal.*

Gentlemen,

To relieve the ſufferings and calamities to which diſeaſe, in its various forms, ſubjects the human race, ſeems to be the moſt illuſtrious ornament we can boaſt of. To attain ſuch an important end, a ſtrict attention to medical diſquiſitions, together with practical inferences, is an eſſential requiſite. Through the medium of your uſeful Journal, we have already experienced the benefit of many intereſting communications. The Vaccine Inoculation, Hydrocephalus, Epilepſy, the Uſe of Digitalis in Pulmonary Conſumption, have all been largely diſcuſſed. I heartily congratulate you upon the high eſtimation your publication ſeems to poſſeſs, and further wiſh it may meet with that general attention its importance ſo juſtly merits.

In retracing the ſubject of epilepſy, I find practitioners ſeem to differ in their opinion reſpecting the ſucceſs of the nitrate of ſilver, as a remedy in that alarming diſeaſe. Some ſpeak of its uſe as nearly approximating to a ſpecific; others, again, ſeem to depreciate its efficacy. There ſeems to be a wiſh for novelty implanted in the human breaſt; conſequently, it is not to be wondered, that when any new remedy is propoſed, that the humane practitioner, not ſatisfied with the former treatment recommended, eagerly embraces the new one, as a probable means of relieving the ſufferings of his patient. This, I apprehend, has been the caſe in Epilepſy; the arg. nit. being ſuppoſed a ſpecific, is indiſcriminately employed; and the practitioner, although fluſhed with the moſt ſanguine hopes of ſucceſs, is frequently diſappointed. Such a want of ſucceſs muſt to every one be particularly obvious. It appears extremely abſurd to imagine that any one medicine, although poſſeſſed of very ſtrong powers, can be adequate to arreſt the progreſs of a diſeaſe depending upon ſuch oppoſite cauſes. It would, I apprehend, be adviſeable to draw a line of diſtinction, and ſpecify in what caſes its adminiſtration ſhould be depended upon, and in what caſes of epilepſy its uſe ſhould be avoided; otherwiſe, the frequent failures ariſing from its general uſe, will ſtrongly militate againſt a remedy which, if judiciouſly employed, will, I flatter myſelf, rank high amongſt thoſe medicines called anti-eleptics, or, more properly, ſuch ſubſtances as have a power of allaying inordinate motions in the body, particularly thoſe irregular contractions which, from peculiar cauſes, take place in the muſcular ſyſtem. I beg leave to ſtate the termination of ſeven epileptic caſes; and, for the ſake of being impartial,

ſhall

ſhall particularize both the ſuccefsful and unſuccefsful ones, as they occurred under the influence of the argentum nitratum.— The medicine was begun in ſmall doſes, and gradually increaſed.

The two out of three unſuccefsful caſes were patients about forty years of age; had been for ſeveral years afflicted with moſt alarming fits, which were frequent in occurrence. They could aſſign no cauſe reſpecting the origin of the complaint. The following particulars were obſervable in theſe two patients: The ſtomach and bowels were much affected with dyſpeptic ſymptoms, great mobility and irritability of the nervous ſyſtem, the tunica albuginea of a white bluiſh colour, attended with a dullneſs of the eye itſelf; the features ſharp, complexion pale, a conſiderable want of expreſſion in the countenance; the apprehenſion dull. The fits were always preceded by general torpor accompanied with vertigo, which were remarkably violent, and attended with an abolition of conſciouſneſs. After the paroxyſm the patients were, for ſeveral days, very languid; and it required a conſiderable time before the aſſociation of ideas could be called regular. It is probable that caſes of this kind depend upon ſome organic affection; and to effect a cure in ſuch caſes, I am afraid is yet beyond the power of the healing art. The third unſuccefsful caſe was a boy about 17, who was for ſeven years ſubject to epilepſy; might not the cauſe here depend upon a crooked ſpine, which was contracted when an infant?

In three out of four caſes, wherein the lunar cauſtic ſeemed advantageous, the patients were about 17 or 18 years old. The fourth, a poor woman, who gave ſuck to a child about a year old. In theſe four patients an aſthenic diatheſis was very conſpicuous; they had not, however, the appearance which was ſo evidently marked in the three unfavourable caſes. The cauſe in theſe four ſeemed to depend upon a want of excitement, induced in conſequence of weak and poor diet. Thus it appears that here debility was the exciting cauſe, the patients from ſome conſtitutional peculiarity being already prediſpoſed. But how, or in what, this peculiarity conſiſts I am totally unable to account for. It has, by writers of conſiderable eminence, been aſſerted that epilepſy from repetition becomes habitual, and, if I may be allowed the expreſſion, a diatheſis epileptica ſeems to take place; to arreſt, however, ſuch catenation, the arg. nitr. ſeems admirably calculated, joined with proper auxillaries, ſuch as diet, exerciſe, &c. And I believe it is only in ſuch caſes as the laſt, wherein we are to place our confidence upon this medicine. Convulſive motions often depend on other cauſes, i. e. worms, teething, accumulation of ſordes in the ſtomach, local irritations, &c. All theſe muſt be treated treated according to circumftances, which can only be judged of by the practitioner at the time of their occurrence.

Thus, Gentlemen, have I ftated, as impartially as poffible, the refult of my fuccefs with the nitrate of filver, and fhould be glad to hear if any of your Correfpondents, through their courfe of practice, fhould meet with a fimilar refult. I forgot to mention, that in the four fortunate cafes, the patients have remained for twelve months free from any return of the fits.

I am extremely forry to find, that in medical inveftigation a degree of refentment feems amongft difputants to predominate over candor and generofity, the true marks of real profeffional worth. And in every fcience where improvement is the aim, we ought invariably to be poffeffed of zeal without prejudice, and faith without fanaticifm. I am, &c.

*Garstang, May* 20, 1801. WILLIAM KNIPE.

---

